# The Primary Care Visit: What Else Could Be Happening?

**DOI:** 10.1155/2012/720506

**Published:** 2012-06-10

**Authors:** Terry Fulmer, Patricia Cabrera

**Affiliations:** Bouve College of Health Sciences, Northeastern University, 360 Huntington Avenue, Boston, MA 02115, USA

## Abstract

The Institute of Medicine Report called for a greater role for nurses within the context of oral health in two recent publications, *Advancing Oral Health in America* (2011) and *Improving Access to Oral Health Care for Vulnerable and Underserved Populations* (2011). Nurses provide care for many vulnerable persons, including frail and functionally dependent older adults, persons with disabilities, and persons with intellectual and developmental disabilities. These persons are the least likely to receive necessary, health-sustaining dental care (which is distinct from mouth care). The mouth, or more accurately, plaque, serves as a reservoir for bacteria and pathogens. The link between mouth care, oral health, and systemic health is well-documented; infections such as pneumonia have been linked to poor oral health. Nurses, therefore, need to reframe mouth care as oral infection control and infection control more broadly. The can provide the preventive measure that are crucial to minimizing systemic infections. Nurses in all settings can potentially provide mouth care, conduct oral health assessments, educate patients about best mouth care practices, and make dental referrals. Yet, nurses are often hesitant to do anything beyond basic oral hygiene—and even in this area, often fail to provide mouth care based on best practices.

## 1. Introduction

The year 2011 was a banner year for oral health reports; the Institute of Medicine published two key reports entitled “*Advancing Oral Health in America*” [1] which outlined the seriousness of poor oral health and lack of access in the oral health care system in America. The second report was entitled “*Improving Access to Oral Health Care for Vulnerable and Underserved Populations*” [2]. The first report was written to describe the aims of oral health as integral to the overall health of the population beyond just dental conditions. It was also a call to arms not only for those in the professional dental community but also for all health care professionals who have interactions with patients. In any given health care encounter, clinicians and in particular nurses have the opportunity to screen for oral cancer, tooth decay, and gum disease and consider any necessary health care referrals as well as provide health education on the role of fluorides, sealants, nutrition, and oral health. Further, the report described how oral diseases can affect other health conditions. There is clear evidence related to the oral-systemic connection and how oral disease can lead to poor health outcomes such as premature birth and cardiac valve disease. The report has seven key recommendations.

1. The secretary of HHS should give the leaders of the new oral health initiative (NOHI) the authority and resources needed to successfully integrate oral health into the planning, programming, policies, and research that occurs across all HHS programs and agencies.
2. All relevant HHS agencies should promote and monitor the use of evidence-based preventative services in oral health, (both the clinical and community based) and counseling across the lifespan.
3. All relevant HHS agencies should undertake oral health literacy and education efforts aimed at individuals and communities and health care professionals.
4. HHS should invest in workforce innovation to improve oral health.
5. CMS should explore new delivery and payment models for Medicare and Medicaid and CHIP to improve access, quality, and coverage of oral health care across the lifespan.
6. HHS should place a high priority on efforts to improve open, actionable, and timely information to advance science and improve oral health through research.
7. To evaluate the NOHI the leaders of the NOHI should convene an annual public meeting of the agency heads to report on the progress of the NOHI [1].

This report, commissioned in 2009 by the United States Department of Health and Human Services, asked the Institute of Medicine to convene a panel to think about strategies for oral health. We have long known that our oral health system is challenged when it comes to access and that many people who encounter a dental visit may, unfortunately, receive less than a comprehensive health assessment while there. The important distinction in the “*Advancing Oral Health in America”* report is that oral health is different from dental health. This change of term reminds all of us that oral health can be addressed by nurses, physicians, social workers, pharmacists, and other allied health professionals who come in contact with patients [1]. By simply asking questions about the patient's oral health, an important difference can be made in the screening and triage of those in need of better oral health care.

## 2. Background and Context

There are numerous reports and ample data to document the need for better access and higher-quality oral health care in America. The Department of Health and Human Services (DHHS) has conducted a number of surveys through the CDC and its National Health and Nutrition Examination Survey (NHANES), in which data are gathered from a sample of the civilian US population for the purpose of better understanding how health and nutrition impact health status outcomes. In 2007, the National Center for Health Statistics (NCHS) compared two reports from NHANES (NHANES III, 1988–1994, and NHANES, 1990–2004), on the dental health of the public. Dye and colleagues have reported optimistic data because in older adults, dental health, specifically edentulism, and periodontitis have improved and the number of dental caries in adolescents decreased from 68% to 59% [3]. According to NHANES data, the dental health of the general adolescent and adult population has improved with a decreased prevalence of dental caries and periodontal disease, likely due to the impact of fluoridation [4]. Only a subset of the adult population, consisting of Mexican-American and non-Hispanic Black men, showed a decrease in oral and dental health in their self-reports, which could be associated with socioeconomic factors. Further, *treatment* of dental decay increased in most ethnic and racial groups, except for non-Hispanic black persons and youths living at or over 200% of the federal poverty level [3]. The IOM report, however, documents a discouragingly high percentage of adults, including older adults who present with untreated dental caries at the time of examination. Finally and alarmingly, there has been an 18–20% reported increase in dental caries in preschool children [1].

Oral health includes dental health and conditions such as cleft palate (1 in 1,000 live births), neoplasms, and neuromuscular or joint disorders of the oral region. Oral cancer has decreased over the past decade, presumably because of a reduction in the use of alcohol and tobacco; however, there is a high mortality because most oral cancers are diagnosed in advanced stages, especially in African Americans, who have a 5-year survival rate of 42% compared to a 63% in whites [1]. In the latest SEER Cancer Statistics Review, oral cancer is among the top 15 cancers [5].

Chevarley has estimated that approximately 1 in 10 persons in the US noninstitutionalized population (approximately 30 million persons) was not able to get or had a delay in accessing needed dental care. Specifically, approximately 5.5 percent of the population was unable to access dental care when needed [6]. The alarming death of Deamonte Driver, a 12-year-old who died in 2007 due to a lack of access to dental care, was a shocking wakeup call for all health care professionals [7].

## 3. The Primary Care Visit: What Else Could Be Happening?

Every primary care visit is an opportunity to provide oral health assessment and care. In the United States, primary care is provided by several categories of health care practitioners including nurse practitioners, family physicians, physician assistants, general internists, pediatricians, and geriatricians. Further, registered nurses, health educators, and medical assistants are an important part of the healthcare workforce who can bring to bear their own knowledge and skills to oral health assessment and care. Private practices, hospital outpatient departments, community health centers, and integrated care systems are all setting for oral health intervention and the mandate to do so is growing [8].

 For example, in 2007 there were 88.9 million visits to hospital outpatient departments (OPDs) in the United States and each encounter, an opportunity for an oral health examination, with 54.7% of those visits to primary care providers [9]. Black or African-American persons (58.4 visits per 100 persons) had higher OPD visit rates. What if each of them received an oral health exam?

## 4. Incentives and Barriers to the Primary Care Oral Examination

 “If you want to provide dental care, go to dental school.” This sentiment is a major barrier to oral health screening for those who are committed to the specialty model of health care. All providers are increasingly under pressure to show productivity in the workplace and argue that the oral examination is not in their scope of practice. Further, there is a concern that the requisite knowledge and skills for the oral health examination are not well understood. Nursing and medical licensing exams require knowledge of oral health, but few medical or nursing schools include oral health in their curriculum [10]. There is an opportunity to fundamentally reconceptualize healthcare delivery practice patterns and interprofessional collaboration as a bidirectional relationship between oral health care and physical health care to improve patient outcomes and access [11, 12]. In 2008, the American Association of Medical Colleges added oral health in their learning objectives but much is to be done before oral health examinations are a standard practice [2]. Only 50% of pediatricians receive oral health training during residency and report a lack of training as a barrier to incorporating oral health in their practices [13]. There are 3.1 million nurses and over 140,000 nurse practitioners in the United States [14] and few have received adequate education related to oral health assessment. While the majority received some instruction related to oral hygiene, few nurses place a high priority on mouth care in the practice setting [11, 15]. In 2006, family medicine residencies included oral health as a requirement; however, only three-quarters of residency directors were aware of this requirement, and in 2006, only two-thirds of the programs were including oral health in the curriculum. These deficiencies in physician training on oral health lead not only to a lack of diagnosis of oral conditions but also to a low rate of referrals to dentists, as 23% of internal medicine residents reported never having referred a patient to the dentist [2].

Educational competencies for nurse practitioners, physicians, and dentists have a great deal in common with opportunities to capitalize on practice expectations. Competencies and learning objectives in the different educational programs overlap. This can be used to promote synergies between educators in the different programs, enriching students' education with knowledge of another discipline which can be incorporated in their future professional practice in benefit of patients [10].

In 2007, the National Hospital Ambulatory Medical Care Survey reported that 55.3% of OPD visits were to a provider other than the patient's primary care provider. It also reported that established patients (those with previous visits to the OPD clinic) made 82.9% of OPD visits and that only 43.1% of visits by these patients were to their primary care provider. The lack in continuity of patients to a constant primary care provider (PCP) may add to the minimization of oral health during examination [9].

It has been described that when used at least twice a year, fluoride varnish reduces dental decay by 38%. In some states, Medicaid reimburses physicians and nurse practitioners $18.18 per visit to apply fluoride varnish 3 times a year to children under 19 years of age [16]. Furthermore, Quiñonez et al. reported that the application of fluoride varnish improves clinical outcomes by 1.52 cavity-free months at a cost of US$7.18 for each cavity-free month gained per child and US$203 for each treatment. These authors have concluded that the use of fluoride varnish in the medical setting is effective in reducing early childhood caries in low-income populations [17]. This is an extremely important data point and adds power to the argument that all of us need to practice oral health assessment and intervention. Despite the evidence that only four percent of pediatricians regularly apply fluoride varnish, there are no data for nurse practitioners [13].

## 5. Barriers to Changing Primary Care Practice Related to Oral Health

There are substantial barriers to changing primary care practice related to oral health. Lack of practitioner confidence, minimal education during formal training, time constraints during the visit, and an absence of referral strategies after examination except in locations where there are dental schools all lead to lack of access and underserved populations. The two recent IOM reports provide specific recommendations that must be addressed and nurse practitioners and physicians are a powerful voice in addressing our current shortfalls in practice [1, 2]. With the looming retirement of the dental workforce [18] there is an urgency to implement some of the creative strategies that are now in pilot phase (Dolce, in press). Our professional practice associations (AMA, ANA) can do much to move the agenda forward by giving voice to the inadequacies of our current educational and payment systems as they relate to oral health.
